# Supplementary material for: Functional Characterization of TetR-like Transcriptional Regulator PA3973 from Pseudomonas aeruginosa
Source: Int J Mol Sci. 2022 Nov 23;23(23):14584. doi: 10.3390/ijms232314584 (PMC9736018; doi:10.3390/ijms232314584)
Supplement: Supplementary file 1 [file ijms-23-14584-s001.zip › Supplementary material PA3973 IJMS.pdf]

Functional characterization of TetR-like transcriptional regulator PA3973 from *Pseudomonas aeruginosa*

Karolina Kotecka, Adam Kawalek, Magdalena Modrzejewska-Balcerek, Jan Gawor, Karolina Zuchniewicz, Robert Gromadka, and Aneta Agnieszka Bartosik *

Institute of Biochemistry and Biophysics, Polish Academy of Sciences, Pawinskiego 5a, 02-106 Warsaw, Poland

* corresponding author's email: anetab2@ibb.waw.pl

SUPPLEMENTARY MATERIALS

Table S1. Bacterial strains, plasmids used and constructed in this study.

strain	description	reference
Escherichia coli		
DH5α	F⁻ Φ80*lacZ*Δ*M15* Δ(*lacZYA-argF*) *U169 recA1 endA1 hsdR17* (r_{K-}, m_{K+}) *phoA supE44 λ thi-1 gyrA96 relA1*	[1]
S17-1	*pro* Δ*hsdR hsdM⁺ recA* Tp^R Sm^R ΩRP4-Tc::Mu Kn::Tn7	[2]
BL21	F⁻ *ompT hsdS*$_B$ ($r_B^-m_B^-$) *gal dcm* (λ DE3)	Novagen
Pseudomonas aeruginosa		
PAO1161	PAO1161 Rif^R *leu*⁻, r⁻, m⁻	[3]
PAO1161	PAO1161 Rif^R r⁻, m⁻	[4]
PAO1161 Δ*PA3973*	PAO1161 Rif^R with deleted gene *PA3973*, allele exchange with the use of pKKB60.6	This study
PAO1161 Δ*PA3972-71*	PAO1161 Rif^R with deleted genes *PA3972* and *PA3971*, allele exchange with the use of pSOB3	This study
**plasmid**	**description**	**reference**
pBBR1-MCS-1	Cm^R, IncA/C broad-host-range cloning vector, *lacZα*–MCS, *mob*, T7p, T3p	[5]
pAMB9.37	pBBR1-MCS-1 derivative with *lacI*^{Q} *tac*p, expression vector	[6]
pABB28.1	pBBR1-MCS-1 derivative with *lacI*^{Q} *tac*p-*flag*, expression vector	[7]
pAKE600	Ap^R, *ori*$_{MB1}$, *oriT*$_{RK2}$, *sacB*, suicide vector	[8]
pKAB240	pUC19 derivative with *his*$_6$-*mcs* (MunI, HindIII, NotI, XhoI, BamHI)-*flag*	[9]
pMEB1	pAMB9.37 derivative with modified *mcs*	[9]
pBGS18	Km^R, *ori*$_{MB1}$, cloning vector	[10]
pET28a(+)	Km^R, *ori*$_{MB1}$, T7p, *lacO*, His$_6$-tag, T7 tag, expression vector	Novagen

pPTO1	Km[R], *oriV*pSC101, promoter-less *xylE* cassette	[11]
pET28mod	Km[R], *ori*MB1, T7p, *lacO*, His_6-tag, modified to remove T7 tag	[12]
pKKB3.11	pAMB9.37 derivative, *lacI*q-*tac*p-*PA3973*, *PA3973* amplified with primers #1/#2 and cloned using EcoRI/SacI	This study
pKKB60.3	pAKE600 derivative with the fragment upstream of *PA3973* amplified with primers #3/#4 and cloned using BamHI/HindIII	This study
pKKB18.3	pBGS18 derivative with the fragment downstream of *PA3973* amplified with primers #5/#6 and cloned using HindIII/EcoRI	This study
pKKB60.6	pAKE600 derivative with fragments upstream and downstream of *PA3973* obtained by re-cloning of *PA3973* downstream region from pKKB18.3 to pKKB60.3 using HindIII/EcoRI	This study
pSOB3.1	pAKE600 derivative with the fragment upstream of *PA3972* amplified with primers #7/#8 and cloned using EcoRI/HindIII	This study
pSOB3.4	pBGS18 derivative with the fragment downstream of *PA3971* amplified with primers #9/#10 and cloned using HindIII/ BamHI	This study
pSOB3	pAKE600 derivative with fragments upstream and downstream of *PA3972-71* obtained by re-cloning of *PA3971* downstream region from pSOB3.4 to pSOB3.1 using HindIII/BamHI	This study
pKKB28.3	pET28mod derivative encoding His_6-PA3973, *PA3973* was amplified with primers #1/#2 and cloned using EcoRI/SacI	This study
pMEB265	pET28mod derivative encoding PA3973-His_6, *PA3973* was amplified with primers #1/#11 and cloned using EcoRI/XhoI	This study
pMEB251	pKAB240 derivative with *PA3973* gene without STOP codon amplified with primers #1/#13 and cloned using	This study

	EcoRI/HindIII	
pMEB255	pMEB1 derivative encoding *lacI*[q]-*tac*p-*PA3973-flag*, *PA3973-flag* re-cloned from pMEB251 using EcoRI/SalI	This study
pMEB267	pPTOI derivative with *PA2468*p-*xylE*, *PA2468*p amplified with primers #19/#20 and cloned using SphI/BamHI	This study
pMEB269	pPTOI derivative with *PA4156*p-*xylE*, *PA4156*p amplified with primers #23/#24 and cloned using SphI/BamHI	This study

Table S2 List of primers used in this study.

nr	name	sequence 5´-3´
#1	3973eF	gcgaattcATGGTCTATCGTGTCACCG
#2	3973eR	gcgagctcTGCAGGTTCATGAGGGTTC
#3	3973mLF	gcggatcCCGGTCAAGTTCGAAGAGTT
#4	3973mLR	gcaagcttAGACCATGACTGAATCCG
#5	3973mPF	gcaagcttAATAGAGGAACCCTCATGAACCTGCAC
#6	3973mPR	gcgaattcCTCCGGCTTGTGCTGGTTGG
#7	3972pF	gcgaattcAGCCAGCGTGAGGTCGATGC
#8	3972upHR	cgcgaagcttCAGGTTCATGAGGGTTCCTC
#9	3972dwHF	cgcgaagcttTGAGGTAACGGGAGAAAAGC
#10	3972dwBR	cgggtaccGGTGACGGTGACGCTGTATTC
#11	3973eR2	gagctcgtgAGGGTTCCTCGCAGA
#12	3973HR	gcaaagcttTGAGGGTTCCTCGCAGACAG
#13	p3973F	gcgaattcGACGGCGTACTGCTCGAC
#14	p3973R*	gcggatccTCACGCTTCAGGCTTTGC
#15	pPA0061F	gcgaattcgCATGCCGGCCCCGTGGACAGCCCGC
#16	pPA0061R	gaggatccGGGGCGGGCTCCGGAGGGT
#17	pPA0195F	gcgaattcgcatgcGATGGGCGGGAATTGTTGG
#18	pPA0195R	gcggatccTCACGAATCTCCTGCGTGA
#19	pPA2468F	gcgaattcgcatgcGGCGCTGGAGATTCCCGGC
#20	pPA2468R	gcggatccCATGGGAAAGTCGGGGCGA
#21	pPA2722F	gagaattcgcatgcCAATGGCGGCGAGGAAAGC
#22	pPA2722R	gcggatccTTCGTGACTCCTTTGCAAG
#23	pPA4156F	gcgaattcgcatgcCACCACGGTTGATCCATAG
#24	pPA4156R	gcggatccAGGATCTTCTCCAAATGGG
#25	pPA4710F	gcgaattcgcatgcGCTCGGCAGGGGAATGGGA
#26	pPA4710R	gaggatccGTGGGACTCCTTGGGTCGG
#27	TproPIIa	CATGTGGTACCATAATAGTTAACGAGAACCCCGG CAGCTGCCGGGGTTATTTTTGGTGGTTCCATGGC
#28	TproPIIb	CATGGCCATGGAACCACCAAAAATAACCCCGGCA GCTGCCGGGGTTCTCGTTAACTATTATGGTACCA
**Primers used in RT-qPCR**		
#29	qPA3973F	GGATCCTGAAGTCGACGAGC
#30	qPA3973R	GAAAGCTGGAATGCGCCAC
#31	qPA3972F	GGGCAAGTACTGGATCTGCA
#32	qPA3972R	CGGAACCTTCCCAGATCGAG
#33	qPA3971F	CAGAACGGCTTCATCCATGC

#34	qPA3971R	TTGAACTCCAGGGTCAGCAC
#35	qPA3970F	AGCGGCAGAACTTCCACTACCC
#36	qPA3970R	GGTGACGGTGACGCTGTATTCG
#37	PA0671qF	GTCGGCGAACTGCAACTAC
#38	PA0671qR	CTGCGGATAGGGTACGTAGG
#39	qPA3614F	AACTCATGCTGCTGGACTCC
#40	qPA3614R	TGGGTATACAGCGGCTTGATG
#41	PA5208qF	CAGGACAAAGTCGCCAATCG
#42	PA5208qR	GAGCATCTGGCCTTGTAGCG
#43	PA5460qF	CTGCCCATCCACATCTCGCC
#44	PA5460qR	AGGTCGTGTTCGGACATTTG
#45	PA5497qF	GGGACAAGAAGTACCGGCTC
#46	PA5497qR	GAGGCCTTGTCCTCGACATC
#47	PA2174qF	AACTGAACCCCGACTTCACG
#48	PA2174qR	GAAGCTGCTGCTCTTCAGGA
#49	D3C65_10195qF	CATTATGGACTTTCGCGCCG
#50	D3C65_10195qR	TTACAGGCGAATGCGACCAC
#51	PROCF	CAGGCCGGGCAGTTGCTGTC
#52	PROCR	GGTCAGGCGCGAGGCTGTCT

Table S3. RNA-seq data for PA3973+ and EV transcriptomes [fold change (FC) ≤ -2 or ≥ 2, FDR adjusted P ≤ 0.01]. Genes identified only in strain PAO1161 but not in PAO1 are described as "not annotated (NA)".

Table S4. Results of ChIP-seq analysis – intergenic regions. 139 PA3973-FLAG ChIP-seq peaks with a fold enrichment (FE) cut-off value of ≥ 2 [FDR<0.01] identified in intergenic regions. RNA-seq data for Δ*PA3973* vs WT strain presented as a fold change (FC) are included. Genes identified only in PAO1161 strain but not in PAO1 are described as "not annotated (NA)".

Table S5. Results of ChIP-seq analysis – coding regions. 179 PA3973-FLAG ChIP-seq peaks identified in coding regions with a fold enrichment (FE) cut-off value of ≥ 2 [FDR<0.01], obtained by the comparison of PA3973-FLAG ChIP samples with negative control samples. RNA-seq data for Δ*PA3973* vs WT strain presented as a fold change (FC) are included. Genes identified only in PAO1161 strain but not in PAO1 are described as "not annotated (NA)".

Table S6. RNA-seq data for transcriptomes of PA3973-deficient cells vs. WT strain [fold change (FC) ≤ -2 or ≥ 2, FDR adjusted P ≤ 0.01]. RNA-seq data for PA3973+ vs EV+ are presented for comparison. Gene identified only in strain PAO1161, but not in PAO1 is described as "not annotated (NA)".

REFERENCES TO SUPPLEMENTARY MATERIAL

1. Hanahan, D. Studies on transformation of *Escherichia coli* with plasmids. *J. Mol. Biol.* **1983**, *166*, 557–580.
2. Simon, R.; O'Connell, M.; Labes, M.; Pühler, A. Plasmid vectors for the genetic analysis and manipulation of *Rhizobia* and other Gram-negative bacteria. *METHODS IN ENZYMOLOGY* **1986**, *118*.
3. Bartosik, A.A.; Mierzejewska, J.; Thomas, C.M.; Jagura-Burdzy, G. ParB deficiency in *Pseudomonas aeruginosa* destabilizes the partner protein ParA and affects a variety of

physiological parameters. *Microbiology (Reading, Engl.)* **2009**, *155*, 1080–1092, doi:10.1099/mic.0.024661-0.
4. Kawalek, A.; Kotecka, K.; Modrzejewska, M.; Gawor, J.; Jagura-Burdzy, G.; Bartosik, A.A. Genome sequence of *Pseudomonas aeruginosa* PAO1161, a PAO1 derivative with the ICEPae1161 integrative and conjugative element. *BMC Genomics* **2020**, *21*, 14, doi:10.1186/s12864-019-6378-6.
5. Kovach, M.E.; Elzer, P.H.; Hill, D.S.; Robertson, G.T.; Farris, M.A.; Roop, R.M.; Peterson, K.M. Four new derivatives of the broad-host-range cloning vector pBBR1MCS, carrying different antibiotic-resistance cassettes. *Gene* **1995**, *166*, 175–176, doi:10.1016/0378-1119(95)00584-1.
6. Ludwiczak, M.; Dolowy, P.; Markowska, A.; Szarlak, J.; Kulinska, A.; Jagura-Burdzy, G. Global transcriptional regulator KorC coordinates expression of three backbone modules of the broad-host-range RA3 plasmid from IncU incompatibility group. *Plasmid* **2013**, *70*, 131–145, doi:10.1016/j.plasmid.2013.03.007.
7. Kotecka, K.; Kawalek, A.; Kobylecki, K.; Bartosik, A.A. The AraC-type transcriptional regulator GliR (PA3027) activates genes of glycerolipid metabolism in *Pseudomonas aeruginosa*. *Int J Mol Sci* **2021**, *22*, 5066, doi:10.3390/ijms22105066.
8. El-Sayed, A.K.; Hothersall, J.; Thomas, C.M. Quorum-sensing-dependent regulation of biosynthesis of the polyketide antibiotic mupirocin in *Pseudomonas fluorescens* NCIMB 10586. *Microbiology (Reading, Engl.)* **2001**, *147*, 2127–2139, doi:10.1099/00221287-147-8-2127.
9. Modrzejewska, M.; Kawalek, A.; Bartosik, A.A. The LysR-type transcriptional regulator BsrA (PA2121) controls vital metabolic pathways in *Pseudomonas aeruginosa*. *mSystems* **2021**, *6*, e0001521, doi:10.1128/mSystems.00015-21.
10. Spratt, B.G.; Hedge, P.J.; te Heesen, S.; Edelman, A.; Broome-Smith, J.K. Kanamycin-resistant vectors that are analogues of plasmids pUC8, pUC9, pEMBL8 and pEMBL9. *Gene* **1986**, *41*, 337–342, doi:10.1016/0378-1119(86)90117-4.
11. Thorsted, P.B.; Shah, D.S.; Macartney, D.; Kostelidou, K.; Thomas, C.M. Conservation of the genetic switch between replication and transfer genes of IncP plasmids but divergence of the replication functions which are major host-range determinants. *Plasmid* **1996**, *36*, 95–111, doi:10.1006/plas.1996.0037.
12. Lukaszewicz, M.; Kostelidou, K.; Bartosik, A.A.; Cooke, G.D.; Thomas, C.M.; Jagura-Burdzy, G. Functional dissection of the ParB Homologue (KorB) from IncP-1 plasmid RK2. *Nucleic Acids Res.* **2002**, *30*, 1046–1055, doi:10.1093/nar/30.4.1046.
